# Molecular Modelling Studies on Pyrazole Derivatives for the Design of Potent Rearranged during Transfection Kinase Inhibitors

**DOI:** 10.3390/molecules26030691

**Published:** 2021-01-28

**Authors:** Swapnil P. Bhujbal, Seketoulie Keretsu, Seung Joo Cho

**Affiliations:** 1 Department of Biomedical Sciences, College of Medicine, Chosun University, Gwangju 501-759, Korea; swapnilbhujbal18@gmail.com (S.P.B.); sekekeretsu@gmail.com (S.K.); 2 Department of Cellular Molecular Medicine, College of Medicine, Chosun University, Gwangju 501-759, Korea

**Keywords:** RET, receptor tyrosine kinases, inhibitors, pyrazole, 3D-QSAR, MM/PBSA

## Abstract

RET (rearranged during transfection) kinase, one of the receptor tyrosine kinases, plays a crucial role in the development of the human nervous system. It is also involved in various cell signaling networks responsible for the normal cell division, growth, migration, and survival. Previously reported clinical studies revealed that deregulation or aberrant activation of RET signaling can cause several types of human cancer. For example, medullary thyroid carcinoma (MTC) and multiple endocrine neoplasia (MEN2A, MEN2B) occur due to sporadic mutation or germline RET mutation. A number of RET kinase inhibitors have been approved by the FDA for the treatment of cancer, such as cabozantinib, vandetanib, lenvatinib, and sorafenib. However, each of these drugs is a multikinase inhibitor. Hence, RET is an important therapeutic target for cancer drug design. In this work, we have performed various molecular modelling studies, such as molecular docking and dynamics simulation for the most active compound of the pyrazole series as RET kinase inhibitors. Furthermore, molecular mechanics Poisson–Boltzmann surface area (MM/PBSA) free energy calculation and 3-dimensional quantitative structure–activity relationship (3D-QSAR) were performed using g_mmpbsa and SYBYL-X 2.1 package. The results of this study revealed the crucial binding site residues at the active site of RET kinase and contour map analysis showed important structural characteristics for the design of new highly active inhibitors. Therefore, we have designed ten RET kinase inhibitors, which showed higher inhibitory activity than the most active compound of the series. The results of our study provide insights to design more potent and selective RET kinase inhibitors.

## 1. Introduction

Receptor tyrosine kinases (RTKs) facilitate communication among cells and their extracellular environment, helping them to carry out important phases in development and maintenance of homeostasis [1]. RTKs comprise an N-terminal extracellular domain (ECD), a transmembrane (TM) domain, an intracellular kinase domain, and, subsequently, a C-terminal tail region [1,2]. There are 58 known RTKs in humans [2], and they share a similar protein structure. RET (rearranged during transfection) kinase is one of the RTKs that is involved in a wide range of complex biological functions, such as cell growth, differentiation, motility, and metabolism [3].

The RET kinase was originally recognized during a human oncogenes screening and has subsequently been associated with numerous human syndromes, like multiple endocrine neoplasia types 2A and 2B, Hirschprung’s, disease and medullary/familial thyroid carcinoma [2]. RET kinase transduces a signal upon activation by ligands of the glial cell line-derived neurotrophic factor (GDNF) family of neurotrophins, which consists of GDNF, artemin (ART), neuturin (NTN), and persephin (PSP). They make a tripartite complex with RET for its activation and a member of extracellular GPI-linked alpha receptors (GFR alpha 1–4). In vitro mutagenesis studies have reported that GFR alpha 1 is responsible for the development of the enteric nervous system (ENS) affected in Hirschsprung’s disease [2,4,5].

There are four key mechanisms for the unusual RET activation in human cancers, including genomic amplification, gain-of-function mutations, chromosomal rearrangements, and autocrine activation [2,6]. Dysregulation of RET signaling plays critical role in different human cancers [7]. RET kinase acts as an ontogenetic driver in various human cancers including papillary and medullary thyroid carcinoma, colorectal carcinoma, lung adenocarcinoma, and salivary gland carcinoma due to the different genetic lesions as gene fusions, point-mutations, and small insertions/deletions [8,9]. Additionally, in other neoplasms, involving breast and pancreatic adenocarcinoma, expression of RET is upregulated [8]. Thus, RET is considered as an important therapeutic target for the treatment of different types of cancer. 

The FDA (Food and Drug Administration) has approved several drugs targeting RET for the treatment of cancer for example, lenvatinib and sorafenib [10,11,12] for differentiated thyroid cancers, and cabozantinib [13] and vandetanib [10,14] for medullary thyroid carcinomas. Many other multikinase inhibitors are in use for the treatment of thyroid or non-thyroid cancers. These include ponatinib [15,16], sunitinib [16], and regorafenib [17]. However, each of these drugs is a multitargeted tyrosine kinase inhibitor that has activity against RET [8]. Hence, there is a need for inhibitors that would specifically inhibit RET kinase. 

Computer-aided drug design (CADD) has emerged as one of the useful techniques for drug design and discovery since the last decade. Although there have been many modeling studies reported on various anticancer derivatives [18,19], the designing of new inhibitors against RET using modeling studies has not been reported before. Therefore, in this study, we have performed various molecular modeling studies on pyrazole derivatives as RET antagonists. The compound **25** of the dataset showed the highest inhibitory activity against RET kinase (pIC_50_ value = 8.8) and was used as a representative compound for the dataset in the study. Molecular docking, molecular dynamics simulation, and MM/PBSA binding free energy calculation were performed for the most active compound of the dataset. Furthermore, 3D-QSAR models were generated to study the structure–activity relation among pyrazole derivatives and, thereby utilizing the structural characteristics studied, a design strategy was developed to design potent anticancer RET agents.

## 2. Results

### 2.1. Molecular Docking

**Figure 1:**
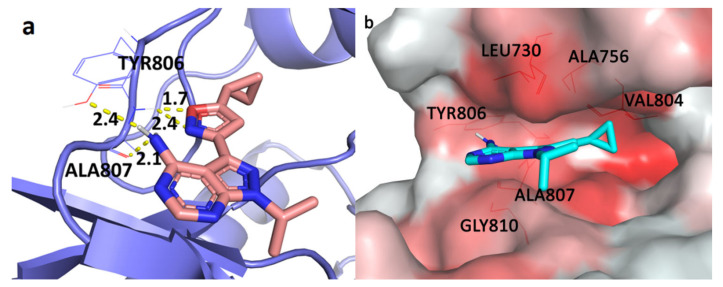
(**a**) Docked conformation of the most active compound **25** (shown in stick model) inside the active site of rearranged during transfection (RET) kinase. Hydrogen bonds are represented as yellow dotted lines and their distances are labeled in angstrom. (**b**) The most active compound **25** (shown in stick model) inside the hydrophobic pocket of RET; the red colored region represents the most hydrophobic surface and white color represents the least hydrophobic surface of the protein. Hydrophobic residues are shown in the red colored line representation.

The docking protocol was assessed by redocking of the co-crystalized ligand into the active site of the RET kinase. The redocked ligand showed similar binding conformation and H-bond interactions to that of co-crystalized ligand, and the root mean square deviation (RMSD) between them was 1.20 Å. This showed that the docking procedure was reliable. The active site of RET kinase is comprised of the residues Leu730, Gly731, Val738, Ala756, Val804, Glu805, Tyr806, Ala 807, Lys808, Tyr809, Gly810, Ser811, and Leu881. The docked conformation of the most active compound **25** was selected based on the binding energy and binding interactions with the active site residues (Figure 1a). Binding energy of the compound **25** with RET kinase was found to be −7.14 kcal/mol with the formation of four H-bonds. Two hydrogen atoms from a fused ring amino group formed two H-bond interactions with the hinge region residue Ala807, which is considered to be important because it mimics the interaction of ATP with kinase. Ala807 was also reported to be an important active site residue in previous X-ray studies of vandetanib, which binds within the ATP binding pocket and forms a H-bond with Ala807 [5]. Furthermore, oxygen and nitrogen atoms from the isoxazole moiety formed 2 H-bond interactions with another hinge region residue Tyr806. 

A python script ‘color h’ was utilized to color hydrophobic residues of the RET kinase and find their interaction with the compound **25**. This script uses Eisenberg hydrophobicity scale (Figure 1b) to color the receptor in PyMOL [20]. It applies red coloring for the most hydrophobic residues, whereas white is used for the least hydrophobic region. Active site residues, which exist in the hydrophobic region (highlighted as lines in the Figure 1b), appear to form hydrophobic interactions with the highly potent compound **25**. The part of ligand with an isoxazole moiety was docked inside the hydrophobic pocket, which formed hydrophobic interactions with residues Leu730, Ala756, Val804, and Tyr806, among which Val804 is a gatekeeper residue [21]. The fused ring was also in a close proximity of hydrophobic residues Ala807, Gly810, Ser811, and Leu881, forming hydrophobic interactions. Hydrophobic interaction with Leu881 has been found to be crucial because it lies within the catalytic spine, which occurs in the surface of the adenine-binding pocket [21]. The selected docked pose of the most active compound was taken as an initial structure to perform molecular dynamics simulation. 

### 2.2. Molecular Dynamics Simulation

**Figure 2:**
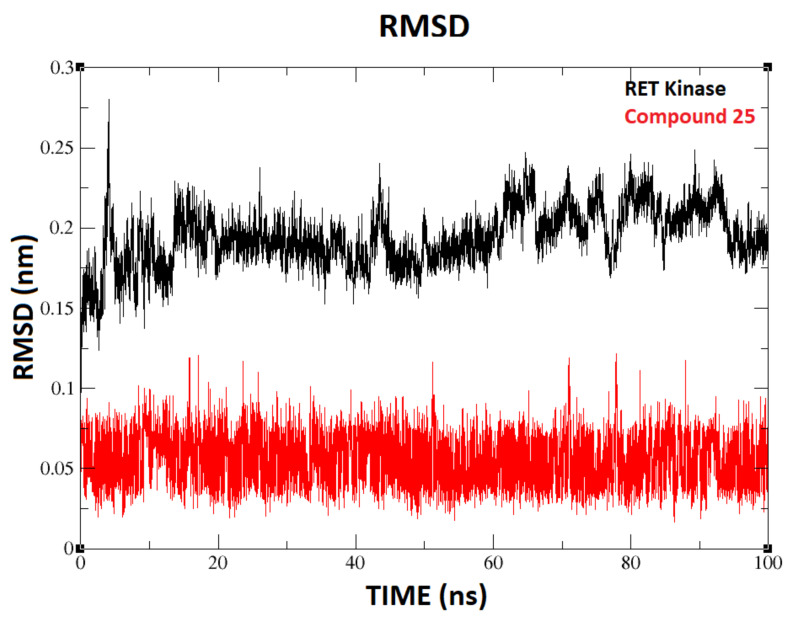
Root mean square deviations (RMSDs) of the protein and compound **25**.

Gromacs-2018 [22] was used to perform MD simulation of the docked complex of compound **25** inside the active site of RET kinase so as to inspect the binding stability and conformation of the ligand. A production run of 100 ns MD simulation was carried out. The root mean square deviation (RMSD) for the ligand and protein were calculated and are shown in Figure 2. The ligand RMSD and protein RMSD are shown in red and black color lines in the graph, respectively. The plot shows that the protein RMSD reached the stability at 20 ns, later fluctuated a bit from 60 to 90 ns, and stabilized at the end of the simulation, which suggests that stable conformation of protein was attained at the end. RMSD of the ligand (compound **25**) stabilized at 10 ns and no fluctuations were observed with less than 0.1 nm deviations until the 100 ns simulation. Minimal fluctuations were observed except for the loop regions during the simulation and the overall fluctuation was less than 2 Å. Overall RMSD analyses indicated that the system was at equilibrium at the end of the simulation. 

**Figure 3:**
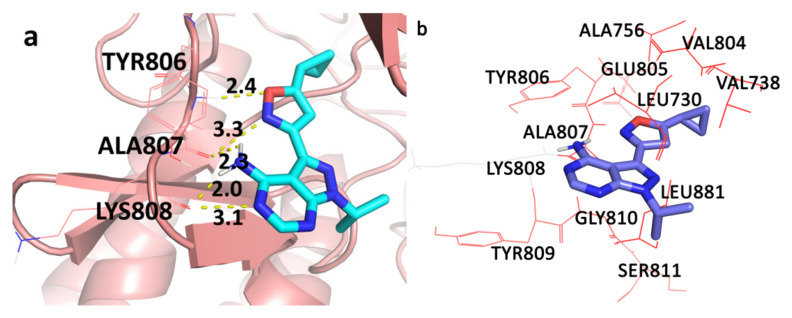
(**a**) 100 ns MD conformation of the most active compound **25** (shown in cyan color and stick model) inside the active site of RET kinase. Hydrogen bonds are represented as yellow dotted lines and their distances are labeled in angstrom. (**b**) The most active compound **25** (shown in stick model) inside the hydrophobic pocket of RET; hydrophobic residues are shown in the red colored line representation.

H-bond analysis of the protein–ligand complex at 100 ns revealed that compound **25** formed 5 H-bond interactions (Figure 3a). Two H-bond interactions were formed with the hinge region residue Ala807, which was consistent with our docking results. The other three H-bonds were formed with the residue Lys808 from the hinge region instead of Tyr806, which was observed in the docking study. The graph of the total number of hydrogen bonds formed between ligand and protein during 100 ns MD simulation is given in Figure S1 of the supplementary material. All the H-bonds were monitored to check their stability throughout the 100 ns MD simulation. Figure S1 revealed that presence of four H-bonds was consistent throughout the simulation. One interaction was lost during the 100 ns simulation; this might be due to the change in the orientation of the isoxazole moiety at the end of the simulation. This change in orientation could be the reason for the formation of H-bonds with residue Lys808 instead of Tyr806. To further explain the same, initial (docked complex) and the final structure (100 ns) of MD were superimposed and are shown in Figure S2 of the supplementary material. In the 100 ns structure, the isoxazole moiety was slightly moved away from its initial docked position causing the change in H-bonds interaction with residue Tyr806, but all the hydrophobic interactions were consistent (shown in Figure 3b).

From overall results, it was observed that H-bond interaction with residue Ala807 and all hydrophobic interactions were consistent throughout the simulation, suggesting the docking procedure was valid and the selected complex was stable. These interactions are crucial in the inhibition of RET kinase. The analyzed MD pose of the most active compound at 100 ns was used as a template in 3D-QSAR study.

### 2.3. MM/PBSA Binding Free Energy Calculation

The MM/PBSA package [23] was used to calculate the binding affinity of compound **25**. The predicted binding free energy was −233.399 kJ/mol. It was combined of Van der Waal energy of −154.682 kJ/mol, electrostatic energy of −28.021 kJ/mol, polar salvation energy of 85.379 kJ/mol and SASA energy of −15.241 kJ/mol. Van der Waals energy as well as nonpolar salvation energy are crucial for the binding of compound **25** with RET kinase. In contrast, polar salvation energy was not favorable for the binding of compound **25**. 

**Figure 4:**
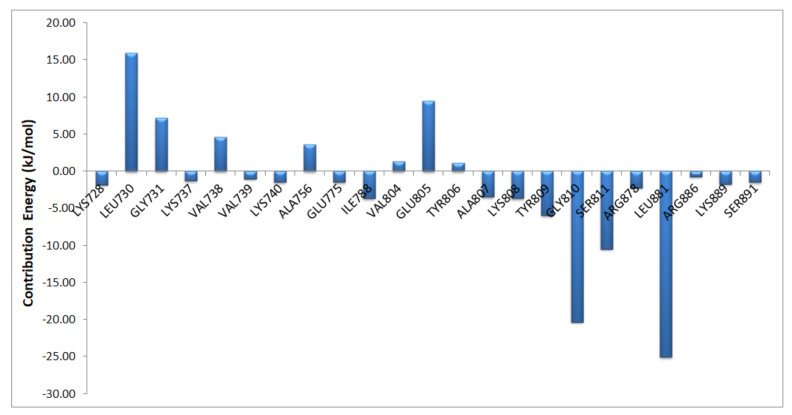
The column chart depicting the each residue contribution in the total binding free energy.

In our docking and MD results, most of the interactions formed by ligand were hydrophobic and were found to be consistent. This explains why the contribution of Van der Waals energy was highest among them. We performed binding free energy decomposition analysis to understand the ligand–protein interactions in detail. The column chart (Figure 4) shows that energy decomposition of each residue. The main contribution to the binding of compound **25** was from residues Leu881, Gly810, Ser811, Ala807, and Lys808, which were involved in the H-bond and hydrophobic interactions. It was also observed that residues Ala756 and Leu730 were in disfavor with the binding of compound **25**. In conclusion, the binding free energy analysis revealed the contribution of important active site residues in the inhibition of RET kinase.

### 2.4. 3D-QSAR

**Figure 5:**
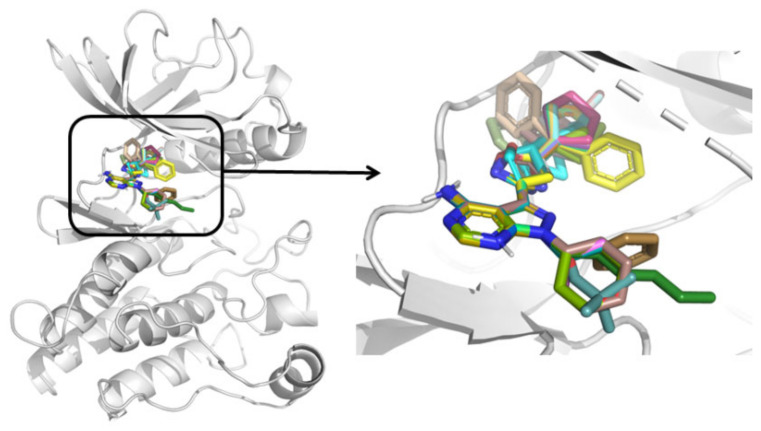
Alignment of the dataset compounds inside the active site of RET kinase (the arrow indicates the enlarged view of the dataset alignment inside the active site of RET kinase).

Receptor-based comparative molecular field analysis (CoMFA) [24,25] and comparative molecular similarity indices analysis (CoMSIA) [26,27] models were developed for the pyrazole derivatives (dataset). All the compounds were sketched and aligned inside the receptor using the MD conformation of the most active compound **25** as a template in SYBYL-X 2.1. The alignment of the dataset compounds is shown in Figure 5. The dataset compounds were divided into training set (27) and test set (8) using the criteria given by Golbraikh et al. and algorithm 4 (activity ranking) was implemented as described in the reported article [28]. We chose algorithm 4 (activity ranking) because there are no large gaps in activity values of dataset compounds and algorithm 4 can construct a test set that represents the whole range of activities. Thus, our test set contains compounds having high, moderate, and low activity (pIC_50_) values. 

It is necessary to calculate various statistical parameters using the partial least square (PLS) method, such as cross-validated correlation coefficient (*q*^2^), non-cross-validated correlation coefficient (*r*^2^), standard error of estimate (SEE), optimal number of components (ONC), and F value to assess the reliability of a 3D-QSAR model. Hence, we derived CoMFA models (*q*^2^ = 0.563, ONC = 6, *r*^2^ = 0.927) for the full dataset and (*q*^2^ = 0.649, ONC = 6, *r*^2^ = 0.955) for the selected test and training sets. The latter model was selected as a final model due to its better *q*^2^ and *r*^2^ values. CoMSIA models were developed using different field combinations and are shown in Table S1 of the Supplementary Material. A combination of electrostatic, hydrophobic, and hydrogen bond acceptor (EHA) fields yielded a CoMSIA model with acceptable statistical values (*q*^2^ = 0.509, ONC = 4, *r*^2^ = 0.745). However, a CoMSIA model generated using an external test set gave better results (*q*^2^ = 0.557, ONC = 5, *r*^2^ = 0.864), which was used for further validation. The detailed statistical values of the chosen CoMFA and CoMSIA models are given in Table 1.

#### Validation of CoMFA and CoMSIA Models

**Figure 6:**
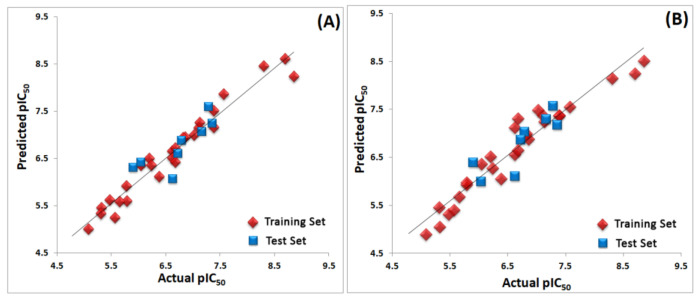
(**A**) Scatter plot for the selected CoMFA model; (**B**) scatter plot for the selected CoMSIA model. The plots shows the actual pIC_50_ versus predicted pIC_50_ activity of the dataset for training and test sets—the training set compounds are represented as diamonds in red color and the test set compounds are represented as squares in blue color. The correlation coefficient (*r*^2^) for CoMFA was 0.955 and for CoMSIA electrostatic, hydrophobic, and hydrogen bond acceptors (EHAs) it was 0.864.

A range of validation techniques were employed to evaluate the predictive ability and the robustness of produced 3D-QSAR models. All the techniques, such as predictive *r*^2^ (external test set), bootstrapping, progressive scrambling (*Q*^2^), and *rm*^2^ metric calculation, exhibited statistical values that were within the acceptable range [29,30,31]. These results proved that the selected models were robust and predictive and their detailed values are shown in Table 1. The experimental and predicted activity values for the established models are presented in Table S2 of the Supplementary Material. The scatter plot for the same is depicted in Figure 6. The compound **25** is shown superimposed with CoMFA and CoMSIA contour maps inside the active site of RET kinase. 

### 2.5. Contour Map Analysis

#### 2.5.1. CoMFA Contour Maps

**Figure 7:**
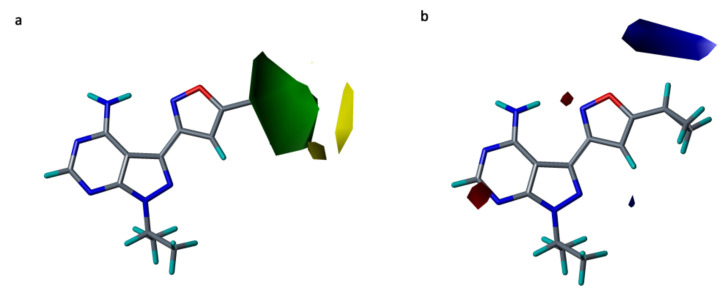
Contour maps for the selected CoMFA model. (**a**) Steric contour map; (**b**) electrostatic contour map—green contour shows the regions favorable for bulky substitutions and yellow contours shows the regions unfavorable for bulky substitutions; Blue contour favors electropositive substitutions while red contour favors electronegative substitutions.

The steric and electrostatic contour maps of CoMFA model are shown in Figure 7a,b, respectively. Favorable regions for steric and electropositive substitutions are denoted by green and blue colors whereas, unfavorable regions for steric and electropositive substitutions are shown by yellow and red colors. 

A big green-colored contour (Figure 7a) was located at R^2^ position of the isoxazole moiety, suggesting that bulky groups are favored at this region to increase the potency. Having a steric group at R^2^ position could interact with many residues of the hydrophobic pocket of RET kinase. This can be explained by the hydrophobic interactions with residues Leu730, Ala756, Val804, and Tyr806, observed in our docking and MD simulation analysis of the most active compound **25**. Similarly, two small yellow colored contours were observed near the R^2^ substitution, which can be position specific because it shows that this region is unfavorable for the bulky groups. This phenomenon could be explained by the positive contributions of residues Leu730, Ala756, and Tyr806, which was revealed in the MM/PBSA binding free energy calculations.

In the electrostatic contour map (Figure 7b), a large blue-colored contour was seen at the R^2^ substitution of the isoxazole ring, which explains that the electropositive group at this position is favorable and it may form H-bond interaction with residue Tyr806. On the other hand, two red colored contours were observed near the R^1^ substitution of a fused ring; suggesting that electronegative groups at this place are favorable. Thus, electronegative substitution at this position might elevate the activity of compounds by forming H-bonds with residues Ala807 and Lys808 of the hinge region. The same interactions were found in our MD simulations study of the highly potent compound **25**. 

#### 2.5.2. CoMSIA Contour Maps

**Figure 8:**
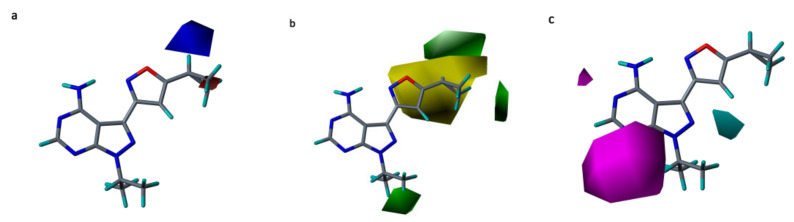
Contour maps for the selected CoMSIA model. (**a**) Electrostatic contour map; (**b**) hydrophobic contour map; (**c**) hydrogen bond acceptor contour map. Blue contour favors electropositive substitutions, while red contour favors electronegative substitutions; green contour shows the regions favorable for hydrophobic substitutions and yellow contours shows the unfavorable regions for bulky substitutions. The favorable region of hydrogen bond acceptor contour map is depicted by magenta color, whereas cyan color denotes that opposite.

The field combination of EHA was used to derive the CoMSIA contour maps and they are shown in Figure 8. We skipped the explanation of CoMSIA electrostatic contour (Figure 8a) since it is similar to the CoMFA electrostatic contour. 

In the hydrophobic contour map (Figure 8b), green- and yellow-colored contours show favorable and unfavorable regions for hydrophobic substitution. Two green colored contours were found at the R^2^ substitution, signifying that the hydrophobic groups at this place are favorable and can form interactions with the residues around. Our docking and MD simulation results of the compound **25** could elucidate this better, as it formed hydrophobic interactions with residues Leu730, Ala756, Val804, and Tyr806. Furthermore, a single green contour was observed near the cyclopropyl moiety of the fused ring (near R^1^ substitution), which indicates that hydrophobic groups in that position could help in increasing the activity of the ligand. The hydrophobic interactions formed by fused ring of the most active compound **25** with active site residues Ala807, Gly810, Ser811, and Leu881 contributed the most in the total binding free energy. This could be the reason why hydrophobic groups are favorable at R^1^ position. 

The favorable region of hydrogen bond acceptor contour map is depicted by magenta color, whereas cyan color denotes that opposite (Figure 8c). Two magenta colored contours were observed near R^1^ position and amino group of fused ring states that presence of hydrogen bond acceptor group in this spot could help in elevating the activity. Compounds with H-bond acceptor at this position could form H-bond with residues Ala807 or Tyr806. This could be confirmed by the H-bond interactions with residues Ala807 and Tyr806 in our docking analysis of the compound **25**. The cyan colored contour near H atom of the isoxazole ring reveals that substituting a hydrogen bond acceptor group at this region decreases the potency.

### 2.6. Designing of RET Kinase Inhibitors

3D-QSAR model development and contour map analysis led us to propose a design strategy to design of the potent compounds. Using this strategy, we designed 10 RET kinase inhibitors and their activity was predicted using the CoMFA model. All designed compounds possessed predicted activity more than the activity of the most active compound of the pyrazole series. The structures and the predicted pIC_50_ values of the designed compounds are presented in Table 2.

Furthermore, we have calculated in silico ADME (absorption, distribution, metabolism, and excretion), physicochemical properties, pharmacokinetics, drug-likeness, and medicinal chemistry friendliness for the designed RET kinase inhibitors using SwissADME web tool (http://www.swissadme.ch/) [32] (Table 3). For lipophilicity, XLOGP3 should be in the range from −0.7 to +6.0. For solubility, log S (calculated with the ESOL model36) should not exceed 6. A qualitative estimation of the solubility class is given according to the following log S scale: insoluble < −10 < poorly < −6 < moderately < −4 < soluble < −2 < very < 0 < highly. The more negative the log Kp (Kp in cm/s), the less skin permeant is the molecule. The synthetic accessibility (SA) score ranges from 1 (very easy) to 10 (very difficult). Drug-likeness evaluates, qualitatively, the chance for a molecule to become an oral drug with respect to bioavailability. Violation to the Lipinski’s rule-of-five filter defined four classes of compounds with probabilities of 11%, 17%, 56%, or 85% [32]. Hence, prediction results depicted in Table 3 show that designed inhibitors possess promising ADME properties. However, experimental testing of the designed RET kinase inhibitors is not possible at this stage in our lab because ours is a bioinformatics modeling lab. 

## 3. Discussion

Various molecular modeling studies were employed in this study to design potent RET kinase antagonists. Molecular docking and MD simulation of the most active compound **25** of the pyrazole series were performed. The results of docking and MD simulation revealed the important active site residues responsible for the inhibition of RET kinase (Figure 3). Most of the hydrophobic and H-bond interactions were consistent in both docking and MD simulation studies, which signified that selected conformation of the most active compound inside the active site of RET was stable and valid for further studies. The selected compound2**5**-RET complex (at 100 ns) from MD simulation was utilized to perform MM/PBSA binding free energy calculation, which showed the residue-wise contribution in the total binding free energy. The binding free energy was found to be −233.399 kJ/mol. Different types of energies were also calculated, such as Van der Waal energy (−154.682 kJ/mol), electrostatic energy (−28.021 kJ/mol), polar salvation energy (85.379 kJ/mol), and SASA energy (−15.241 kJ/mol). Among all, Van der Waal’s energy contributed the most to total binding free energy. This could be the reason why all the hydrophobic interactions observed in our docking study were consistent with MD simulation results. Hydrophobic residues Leu881, Gly810, Ser811, Ala807, and Lys808 were found to be important, which could be verified by the column chart of active site residue contribution in the binding free energy (Figure 4). The residues that were observed in our study were also reported to be important for the RET kinase inhibition in previous experimental and modeling studies. After understanding the important residues required to inhibit the RET kinase, we performed a structure–activity relationship study (CoMFA and CoMSIA) of pyrazole derivatives. We obtained statistically reasonable CoMFA and CoMSIA (EHA) models and validated these using different validation methods to check their reliability and predictive ability (Table 1). The bootstrapping, external test set, progressive scrambling, and *rm*^2^ metric calculation analysis showed that models were reliable and predictive. The contour map analysis of CoMFA and CoMSIA revealed the structural modifications required at R^1^ and R^2^ positions to increase the activity (Figure 7 and Figure 8). Hydrogen bond acceptor and electronegative groups were found to be favorable at R^1^ substitution, whereas electropositive, steric, and H-bond donor groups were found to be favorable at R^2^ substitution to increase the potency of inhibitors. Using this structural knowledge, we have designed 10 RET kinase inhibitors, which showed predicted activity more than the most active compound **25** of the pyrazole series (Table 2). Hence, overall outcome of our study can help modelers and medicinal chemists to design and synthesize potent RET kinase inhibitors.

## 4. Materials and Methods

### 4.1. Test Set/Training Set Selection for 3D-QSAR Analyses

A dataset of 35 RET kinase inhibitors, with the pyrazole ring as a common scaffold, was taken for our study [7,9]. SYBYL-X 2.1 was utilized to draw and optimize the structures using energy minimization with Tripos force field [33]. Biological activities (IC_50_) were converted into pIC_50_ (−log IC_50_) values and were implemented as dependent variables to generate the 3D-QSAR models. The activity log span of pIC_50_ values of inhibitors was more than 3 logarithmic units, which lay within the prerequisite range [34]. The dataset was separated into a training set of 27 compounds for model generation and 8 compounds as test set for model validation based on the activity span of compounds. The chemical structures of the dataset compounds with their IC_50_ values are listed in Table 4 where the test set compounds are denoted by *.

### 4.2. Modeling of the Missing Residues

The crystal structure of RET kinase with high resolution (PDB ID: 4CKJ) was obtained from the protein data bank for our study [35]. It contains missing residues in the loop region from residue Gly823 to Glu843, which were modeled and refined using the modellerV9.14 [36]. The final modeled structure was selected depending on the energy, GA341 score [37], and DOPE score [38].

### 4.3. Preparation of the Protein and Molecular Docking 

We used Autodock 4 to perform molecular docking of the most active compound **25** of the series [39,40]. The crystal structure (PDB: 4CKJ) was utilized as reference to dock the compound **25** inside the active site of the RET kinase. Prior to the docking, the receptor structure was prepared by the addition of polar hydrogens, applying Kollman charges and assigning AD4 atom types. Consequently, Autodock tools were used to prepare the ligand by keeping the number of rotatable bonds less than 6. The active site grid was generated using the x, y, and z coordinates of the active site. The grid box was extended to 70 × 70 × 70 points, with a grid spacing of 0.375 Å. The docking was executed using the Lamarckian genetic algorithm (LGA) by setting the number of the genetic algorithm (GA) run to 100. The docked pose of compound **25** was selected based on its interactions with RET kinase and the lowest binding energy.

### 4.4. Molecular Dynamics Simulations

The MD simulation was performed using Gromacs-2018 [41]. The protein and ligand topology files were generated using Amber99SB force field [41] and general AMBER force field (GAFF) [42], respectively. The ligand force field parameters were generated using the ACPYPE program [43]. The system was neutralized by adding eight chloride ions. A three-point water model (TIP3P) was used as the solvent. Energy minimization was done by using the steepest descent method for 50,000 steps. Subsequently, the system was equilibrated first via a NVT ensemble for a 100 ps at 300 K using Berendsen thermostat [44] and then using NPT for 100 ps with the constant pressure of 1 atm. The bonds were constrained using the LINCS algorithm [45]. The particle mesh Ewald (PME) method [46] was used to handle the long-range coulombic interactions. A 100 ns production run was performed using NPT ensemble at 300 K with 1.0 atm pressure with a time step of 2 fs. 

### 4.5. MM/PBSA Binding Free Energy Calculations 

The g_mmpbsa package was employed to execute molecular mechanics Poisson–Boltzmann surface area (MM/PBSA) free energy calculation [23]. The last 1 ns from the production run of 100 ns MD simulation was utilized for the calculation of binding free energy. The binding free energy comprises of three energetic terms, including potential energy in vacuum, polar-solvation energy, and nonpolar solvation energy. The molecular mechanics force field parameters were used to calculate both bonded (angle, bond, and dihedral) and nonbonded (electrostatic and van der Waal) interactions included in the potential energy in vacuum. Similarly, the Poisson–Boltzmann equation and solvent accessible surface area (SASA) model was used to calculate polar solvation energy and nonpolar solvation energy, respectively [47,48]. The estimation of binding free energy for the protein-ligand complex in a solvent was calculated based on the equation given below:

ΔG_binding_ = ΔG_complex_ − (ΔG_protein_ + ΔG_ligand_) (1)

where, ΔG_binding_ is the binding free energy and ΔG_complex_, ΔG_protein_, and ΔG_ligand_ represent the free energy of complex, protein, and ligand, respectively. 

### 4.6. Receptor-Based CoMFA and CoMSIA Models 

3D-QSAR models; comparative molecular field analysis (CoMFA) and comparative molecular similarity indices analysis (CoMSIA) were established to correlate 3D structures of ligands and the biological activity using SYBYL-X2.1 [24,25]. The dataset compounds were aligned inside the receptor using the distill rigid alignment method and most active compound (compound **25**) as a template. The models were developed for the same training and test set. The CoMFA model was generated using steric and electrostatic fields, which are calculated using Lennard-Jones and Coulombic potentials [25]. Whereas the CoMSIA model utilized fields such as steric, electrostatic, hydrophobic, hydrogen bond acceptor, and hydrogen bond donor [24]. Cross-validated leave-one-out (LOO) and a non-cross-validation partial least squares (PLS) analysis were used to obtain the 3D-QSAR models. The correlation coefficient (*r*^2^) was calculated using the formula given below:

(1) $$r^{2}=1-[\sum (y-\bar{y}){}^{2}/\sum (y-\hat{y}){}^{2}]$$

where, y is the observed response variable, $\hat{y}$ is its mean, and $\bar{y}$ is the corresponding predicted value. Statistical values of *q^2^, r^2^*, standard error of estimate (SEE), and F values were used to evaluate and select the final models. CoMSIA models were developed with different field combinations and the one with acceptable *q*^2^ and *r*^2^ values were selected. The robustness and predictive ability of the models were validated using various validation techniques such as bootstrapping, progressive scrambling, predictive *r*^2^ and *rm*^2^ metric calculations. 

#### 3D-QSAR Model Validation

CoMFA and CoMSIA models were assessed for the predictive ability using various validation techniques. All the models are examined for stability and robustness with external test set validation, a 100 run of bootstrapping, progressive sampling, and predictive *r*^2^ and *rm*^2^ metric calculations. Then, 100 runs with 2 to 10 bins of the progressive scrambling were performed to validate the models [49]. Lastly, 3D-QSAR outcomes were graphically denoted by field contour maps using the field type ‘StDev*Coeff’.

## 5. Conclusions

RET kinase is a one of the important receptor tyrosine kinases that play crucial role in cell division, development, and maturation and it is involved in many types of human cancer. Hence, it makes RET an ultimate drug target. In our study, we have utilized various modeling techniques, like molecular docking, MD simulation, and MM/PBSA binding free energy calculation, in order to investigate and find the crucial active site residues responsible for the inhibition of RET kinase. The overall analysis revealed that active site residues Ala807, Lys808, Gly810, Ser811, and Leu881 were important for the RET inhibition. The residues Gly810, Ser811, and Leu881 were found to contribute more to the total binding energy. Furthermore, CoMFA and CoMSIA (EHA) resulted in reasonable statistical models in terms of *q*^2^ and *r*^2^. The models were found to be predictive and reliable. Analysis of contour maps developed using selected 3D-QSAR models was consistent with our docking and MD results, thereby it explicated the structural characteristics required to design more potent inhibitors. Using this information, we designed 10 RET kinase inhibitors. Our designed RET inhibitors showed predicted activity greater than the most potent compound of the pyrazole series, which can be further evaluated using experimental studies for their specific contribution in the inhibition of RET kinase. 

## Figures and Tables

**Table 1:** Detailed statistical values of the selected comparative molecular field analysis (CoMFA) and comparative molecular similarity indices analysis (CoMSIA) models.

Parameter	Full Model		Test Set 11	
	CoMFA	CoMSIA (EHA)	CoMFA	CoMSIA (EHA)
*q* ^2^	0.563	0.509	0.649	0.557
ONC	6	4	6	5
SDEP	0.666	0.682	0.679	0.744
*r* ^2^	0.927	0.745	0.955	0.864
SEE	0.272	0.491	0.242	0.412
F value	59.187	21.918	71.390	26.749
BS-*r*^2^	-	-	0.982	0.945
BS-SD	-	-	0.013	0.038
*Q* ^2^	-	-	0.531	0.508
*r* ^2^ *_pred_*	-	-	0.652	0.660
*rm* ^2^	-	-	0.532	0.601
*Delta* rm^2^	-	-	0.073	0.072

*q*^2^: squared cross-validated correlation coefficient; ONC: optimal number of components; SDEP: standard error of prediction; *r*^2^: squared correlation coefficient; SEE: standard error of estimation; F value: F-test value; BS-*r*^2^: bootstrapping *r*^2^ mean; BS-SD: bootstrapping standard deviation; *Q*^2^: progressive sampling; *r*^2^*_pred_*: predictive *r*^2^, *rm*^2^: average *rm*^2^ for the dataset; *Delta rm*^2^: *Delta rm*^2^ for the dataset.

**Table 2:** The structures and the predicted pIC_50_ values of the designed RET kinase antagonists.

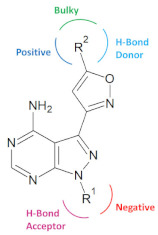			
**Compound**	**R^1^**	**R^2^**	**pIC_50_**
D1	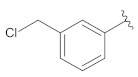	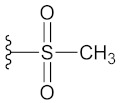	10.31
D2	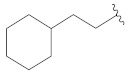	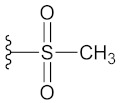	8.85
D3	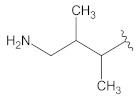	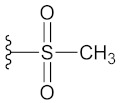	8.97
D4	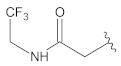	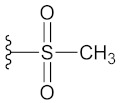	9.02
D5	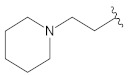	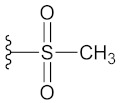	10.26
D6	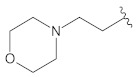	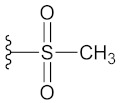	10.74
D7	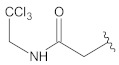	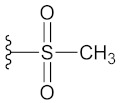	9.08
D8	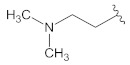	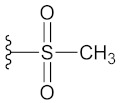	9.20
D9	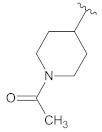	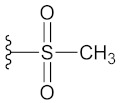	9.22
D10	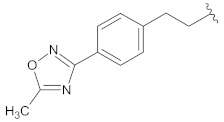	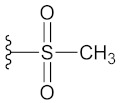	8.80

Here, D1 to D10 indicate designed compounds **1** to **10** (D: designed compound).

**Table 3:** In silico (absorption, distribution, metabolism, and excretion (ADME) prediction and synthetic accessibility values of new designed RET kinase antagonists.

Designed Compound	Lipophilicity	Water Solubility		Pharmacokinetics		Synthetic Accessibility	Druglikeness
	Log *P_o/w_*	Log *S* (ESOL)	Class	GI Absorption	Log K_p_ (Skin Permeation)		Lipinski Rule
D1	1.72	−3.81	Moderately soluble	Low	−7.46 cm/s	3.86	Yes; 0 violation
D2	1.97	−4.10	Moderately soluble	Low	−6.68 cm/s	4.28	Yes; 0 violation
D3	1.08	−2.19	Soluble	Low	−8.54 cm/s	4.57	Yes; 0 violation
D4	0.48	−2.58	Soluble	Low	−8.61 cm/s	3.94	Yes; 1 violation: NorO > 10
D5	1.51	−2.71	Soluble	Low	−8.26 cm/s	4.21	Yes; 0 violation
D6	1.99	−1.96	Soluble	Low	−9.13 cm/s	4.13	Yes; 1 violation: NorO > 10
D7	0.86	−3.35	Moderately soluble	Low	−8.39 cm/s	3.96	Yes; 1 violation: NorO > 10
D8	1.26	−1.98	Soluble	Low	−8.61 cm/s	3.99	Yes; 0 violation
D9	0.47	−2.31	Soluble	Low	−8.96 cm/s	4.12	Yes; 1 violation: NorO > 10
D10	2.43	−4.14	Moderately soluble	Low	−7.75 cm/s	4.31	Yes; 0 violation

Where, Log *P*_o/w_: partition coefficient between *n*-octanol and water; Log *S* (ESOL): decimal logarithm of the molar solubility in water; Log K_p_: the skin permeability coefficient.

**Table 4:** The chemical structures of the pyrazole derivatives with their IC_50_ and pIC_50_ values.

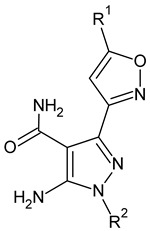 Compounds **2**–**14**		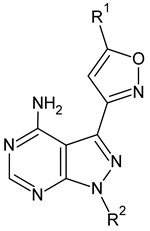 Compounds **18**–**35**		
**Compound**	**R^1^**	**R^2^**	**IC_50_ (µM)**	**pIC_50_**
**1**	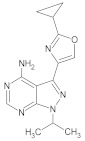		0.139	6.857
2 *	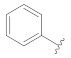	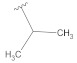	0.236	6.627
**3**	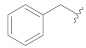	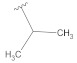	1.639	5.785
**4**	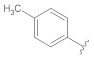	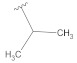	1.661	5.780
**5**	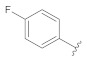	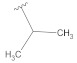	0.416	6.381
**6**	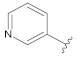	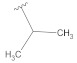	3.395	5.469
**7**	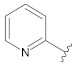	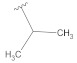	4.803	5.318
**8**	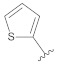	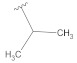	0.078	7.108
9 *	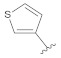	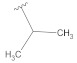	0.162	6.790
**10**	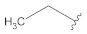	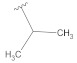	0.631	6.200
11 *	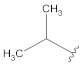	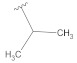	1.259	5.900
**12**	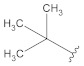	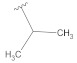	2.224	5.653
13 *	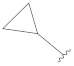	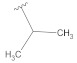	0.044	7.357
**14**	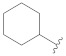	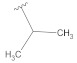	2.695	5.569
**15**	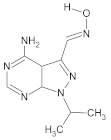		4.90	5.310
**16**	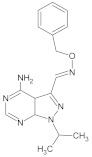		8.33	5.079
**17**	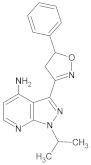		0.583	6.234
18 *	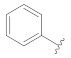	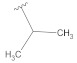	0.190	6.721
**19**	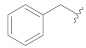	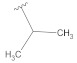	0.241	6.618
**20**	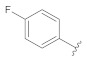	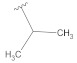	0.150	6.824
21 *	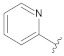	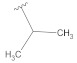	0.910	6.041
**22**	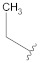	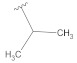	0.041	7.387
**23**	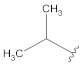	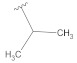	0.041	7.387
**24**	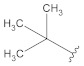	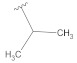	0.210	6.678
**25**	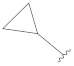	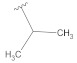	0.0014	8.854
**26**	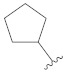	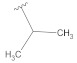	0.241	6.618
**27**	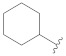	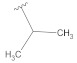	0.910	6.041
**28**	H	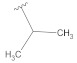	0.213	6.672
29 *	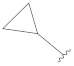	H	0.052	7.284
**30**	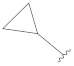	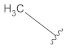	0.027	7.569
**31**	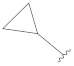	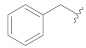	0.074	7.131
**32**	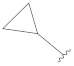	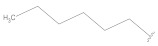	0.096	7.018
33 *	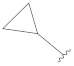	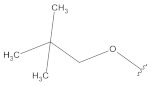	0.068	7.167
**34**	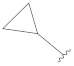	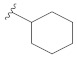	0.002	8.699
**35**	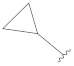	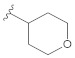	0.005	8.301

* Test set compounds.

## Data Availability

The data presented in this study are available in the supplementary material.

## Supplementary Materials

The following are available online. Table S1: CoMSIA models developed using different combinations of fields. Table S2: Residual values of the selected CoMFA and CoMSIA models. Figure S1: The graph of the number of hydrogen bonds during 100 ns MD simulation. Figure S2: Superimposition of the initial (docked complex) and the final (100 ns) MD structure of the compound **25** inside in the binding site of RET kinase.
