# Supplementary material for: Molecular Modelling Studies on Pyrazole Derivatives for the Design of Potent Rearranged during Transfection Kinase Inhibitors
Source: Molecules. 2021 Jan 28;26(3):691. doi: 10.3390/molecules26030691 (PMC7865942; doi:10.3390/molecules26030691)

Molecular modelling studies on pyrazole derivatives for the design of potent Rearranged during Transfection kinase inhibitors

Swapnil P. Bhujbal[1] · Seketoulie Keretsu[1] · Seung Joo Cho[1,2,*]

[1]*Department of Biomedical Sciences, College of Medicine, Chosun University, Gwangju 501-759, Republic of Korea*

[2]*Department of Cellular·Molecular Medicine, College of Medicine, Chosun University, Gwangju 501-759, Republic of Korea*

Supplementary Materials

Tables

Table S1. CoMSIA models developed using different combinations of fields.

CoMSIA	q^2	ONC	SEP	r^2	SEE	F value	Percentage contribution				
							S	E	H	A	D
S	0.332	6	0.823	0.767	0.437	15.320	100	-	-	-	-
E	0.394	6	0.784	0.595	0.641	6.850	-	100	-	-	-
H	0.297	2	0.790	0.813	0.435	20.310	-	-	100	-	-
A	0.340	3	0.778	0.564	0.665	6.034	-	-	-	100	-
D	0.204	2	0.840	0.446	0.750	3.752	-	-	-	-	100
SE	0.415	6	0.770	0.850	0.390	26.38	59.5	40.5	-	-	-
EH	0.467	5	0.722	0.780	0.464	20.556	-	33.6	66.4	-	-
EA	0.444	6	0.751	0.633	0.610	8.047	-	55.1	-	44.9	-
ED	0.368	6	0.801	0.625	0.617	7.763	-	76.9	-	-	23.1
SH	0.313	2	0.781	0.572	0.617	21.365	29.1	-	70.9	-	-
SA	0.406	2	0.726	0.623	0.579	26.442	39.3	-	-	60.7	-
SD	0.300	2	0.788	0.761	0.493	14.829	76.3	-	-	-	23.7
HA	0.434	3	0.720	0.845	0.397	25.366	-	-	69.9	30.1	-
HD	0.341	2	0.765	0.806	0.443	19.408	-	-	84.6	-	15.4
SEH	0.436	5	0.744	0.805	0.437	23.907	22.2	36.8	41.0	-	-
SEA	0.469	4	0.709	0.738	0.498	21.137	32.5	35.9	-	31.7	-

SED	0.420	4	0.741	0.715	0.520	18.772	36.7	36.6	-	-	26.7
**EHA**	**0.509**	**4**	**0.682**	**0.745**	**0.491**	**21.918**	**-**	**28.1**	**44.2**	**27.7**	**-**
EHD	0.465	5	0.724	0.798	0.445	22.880	-	32.3	48.9	-	18.8
SHA	0.403	2	0.728	0.623	0.579	26.427	20.7	-	46.5	34.8	-
SHD	0.360	2	0.754	0.540	0.639	18.809	18.4	-	37.2	-	44.4
EAD	0.386	5	0.725	0.622	0.609	9.539	-	43.5	-	33.1	23.4
HAD	0.397	3	7.444	0.669	0.551	20.915	-	-	52	26.5	21.5
SEHD	0.469	6	0.734	0.855	0.383	27.566	23.7	23.2	36.5	-	16.6
SEHA	0.471	4	0.708	0.767	0.470	24.688	19.0	25.4	32.4	23.2	-
SEAD	0.458	4	0.717	0.818	0.435	20.408	43.2	25.1	-	16.1	15.5
EHAD	0.478	4	0.703	0.739	0.499	21.022	-	23.9	40	19.8	16.3
SHAD	0.387	3	0.749	0.677	0.544	21.666	21.5	-	36.6	21.4	20.5
SEHAD	0.481	3	0.689	0.727	0.500	27.531	17	18.1	29.1	17.5	18.4

Table S2. Residual values of the selected CoMFA and CoMSIA models.

**Compound**	**CoMFA**			**CoMSIA (EHA)**		
	**Actual pIC_{50}**	**Predicted pIC_{50}**	**Residual**	**Actual pIC_{50}**	**Predicted pIC_{50}**	**Residual**
1	6.857	6.956	-0.099	6.857	6.869	-0.012
2	6.627	6.065	0.562	6.627	6.113	0.514
3	5.785	5.599	0.186	5.785	5.97	-0.185
4	5.780	5.919	-0.139	5.78	5.921	-0.141
5	6.381	6.113	0.268	6.381	6.047	0.334
6	5.469	5.625	-0.156	5.469	5.308	0.161
7	5.318	5.458	-0.140	5.318	5.05	0.268
8	7.108	7.149	-0.041	7.108	7.371	-0.263
9	6.790	6.878	-0.088	6.79	7.041	-0.251

10	6.200	6.499	-0.299	6.2	6.51	-0.31
11	5.900	6.306	-0.406	5.9	6.402	-0.502
12	5.653	5.591	0.062	5.653	5.675	-0.022
13	7.357	7.244	0.113	7.357	7.185	0.172
14	5.569	5.252	0.317	5.569	5.392	0.177
15	5.310	5.334	-0.024	5.31	5.453	-0.143
16	5.079	5.014	0.065	5.079	4.889	0.19
17	6.234	6.354	-0.120	6.234	6.265	-0.031
18	6.721	6.607	0.114	6.721	6.879	-0.158
19	6.618	6.659	-0.041	6.618	6.552	0.066
20	6.824	6.943	-0.119	6.824	6.98	-0.156
21	6.041	6.417	-0.376	6.041	6.005	0.036
22	7.387	7.156	0.231	7.387	7.371	0.016
23	7.387	7.506	-0.119	7.387	7.359	0.028
24	6.678	6.716	-0.038	6.678	6.638	0.04
25	8.854	8.231	0.623	8.854	8.506	0.348
26	6.618	6.507	0.111	6.618	7.113	-0.495
27	6.041	6.357	-0.316	6.041	6.352	-0.311
28	6.672	6.429	0.243	6.672	7.304	-0.632
29	7.284	7.595	-0.311	7.284	7.583	-0.299
30	7.569	7.858	-0.289	7.569	7.544	0.025
31	7.131	7.257	-0.126	7.131	7.237	-0.106
32	7.018	6.993	0.025	7.018	7.476	-0.458
33	7.167	7.073	0.094	7.167	7.305	-0.138
34	8.699	8.608	0.091	8.699	8.243	0.456
35	8.301	8.457	-0.156	8.301	8.145	0.156

Figures

Figure S1. The graph of the number of hydrogen bonds during 100 ns MD simulation.

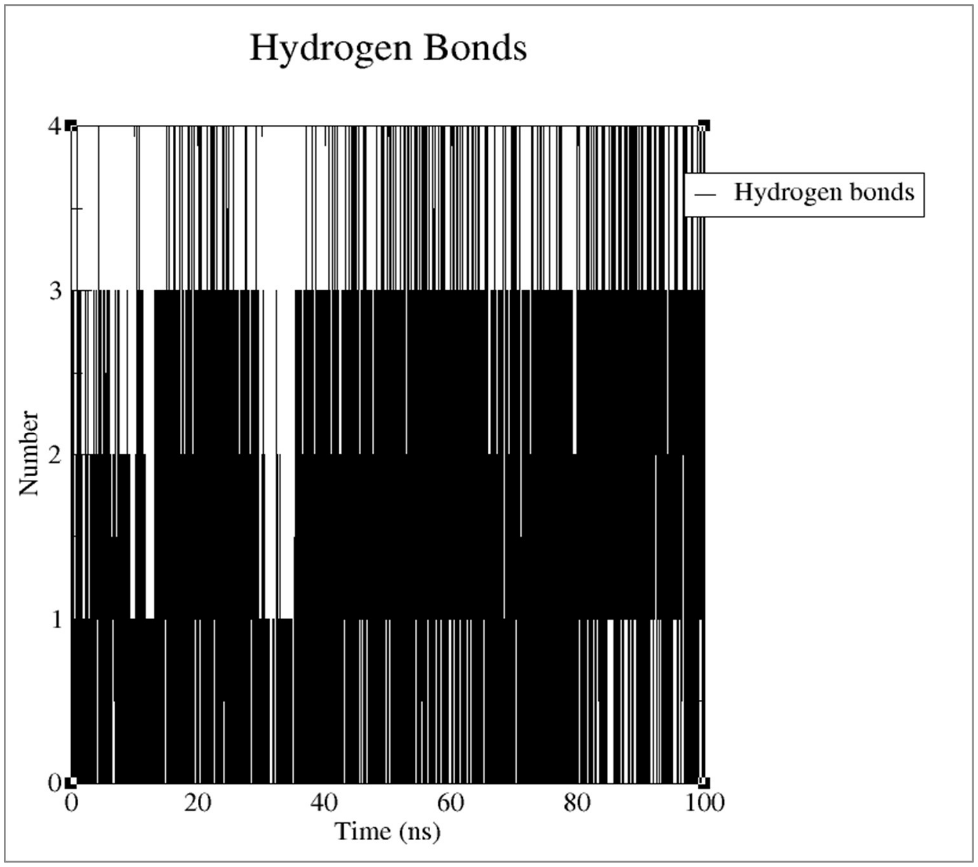

Figure S2. Superimposition of the initial (docked complex) and the final (100 ns) of MD structure of the compound 25 inside in the binding site of RET kinase. (Purple color shows the docked complex and salmon color denotes the 100 MD complex)

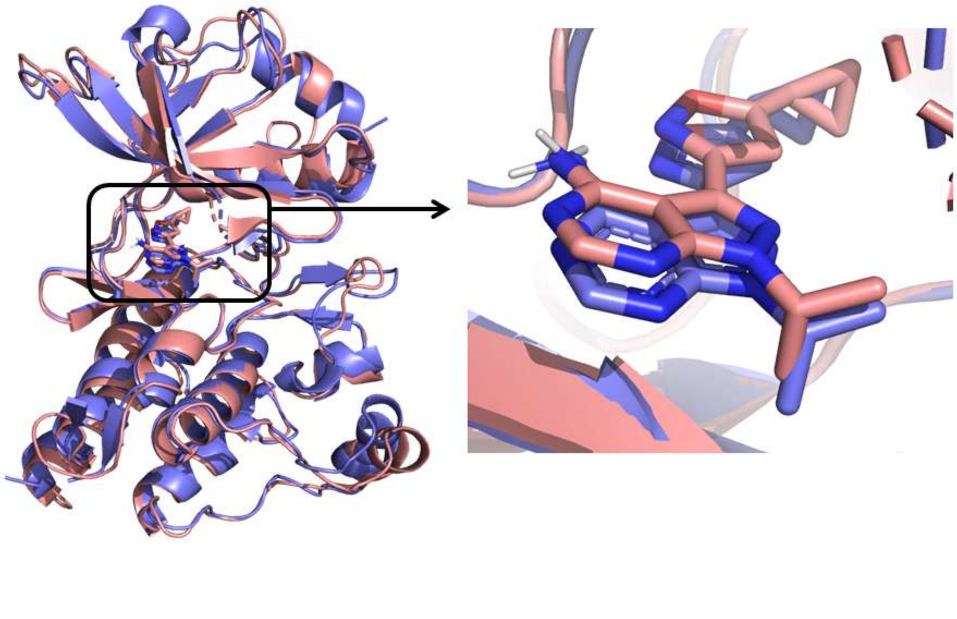

Supplement: Supplementary file 1 [file molecules-26-00691-s001.pdf]
